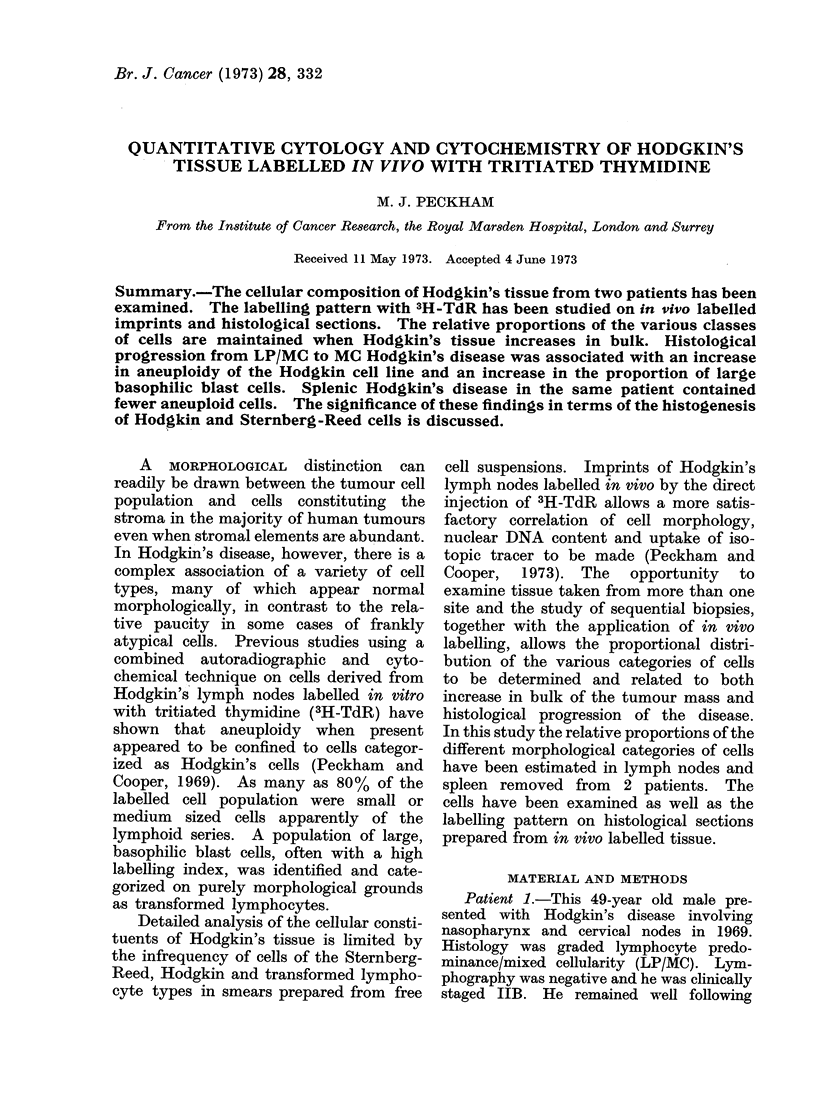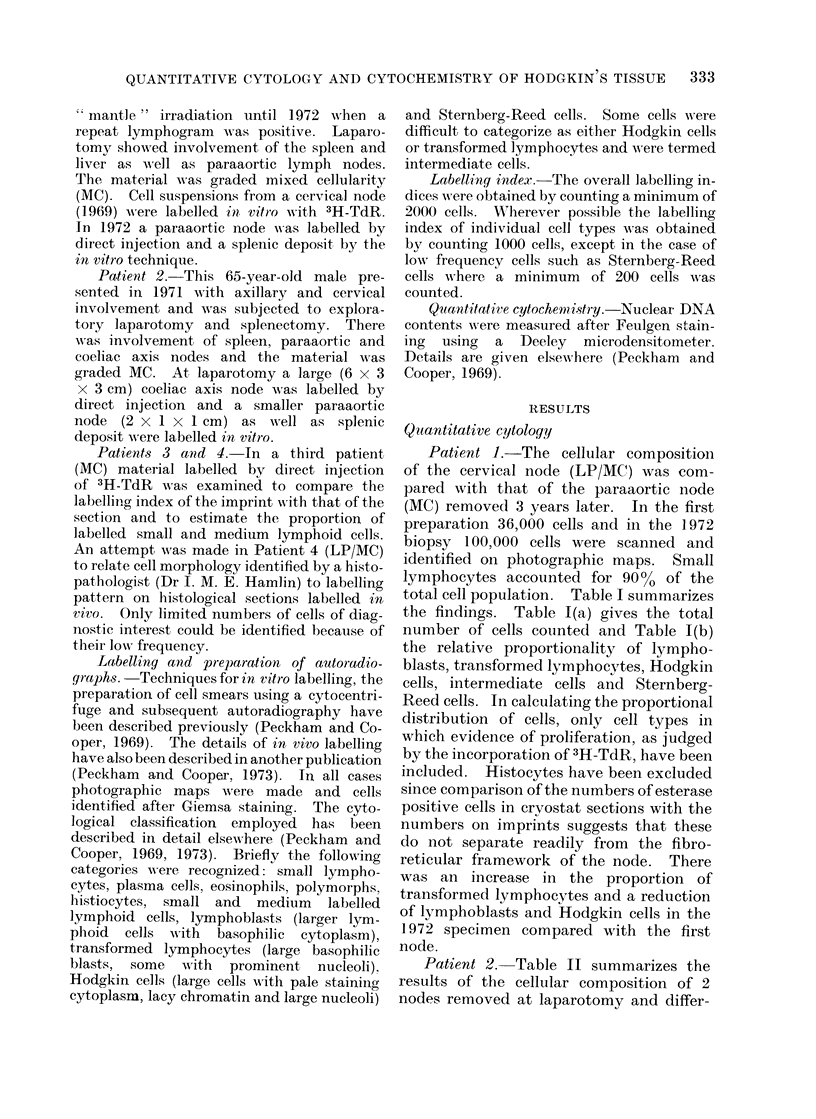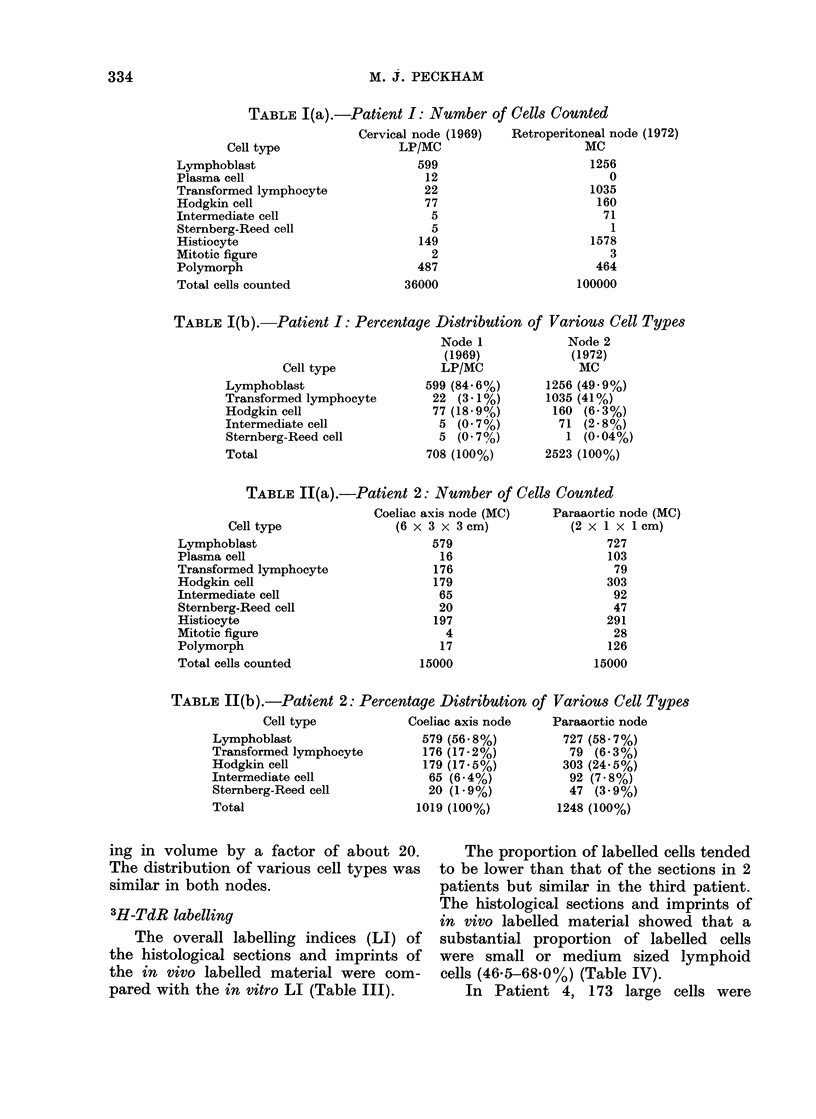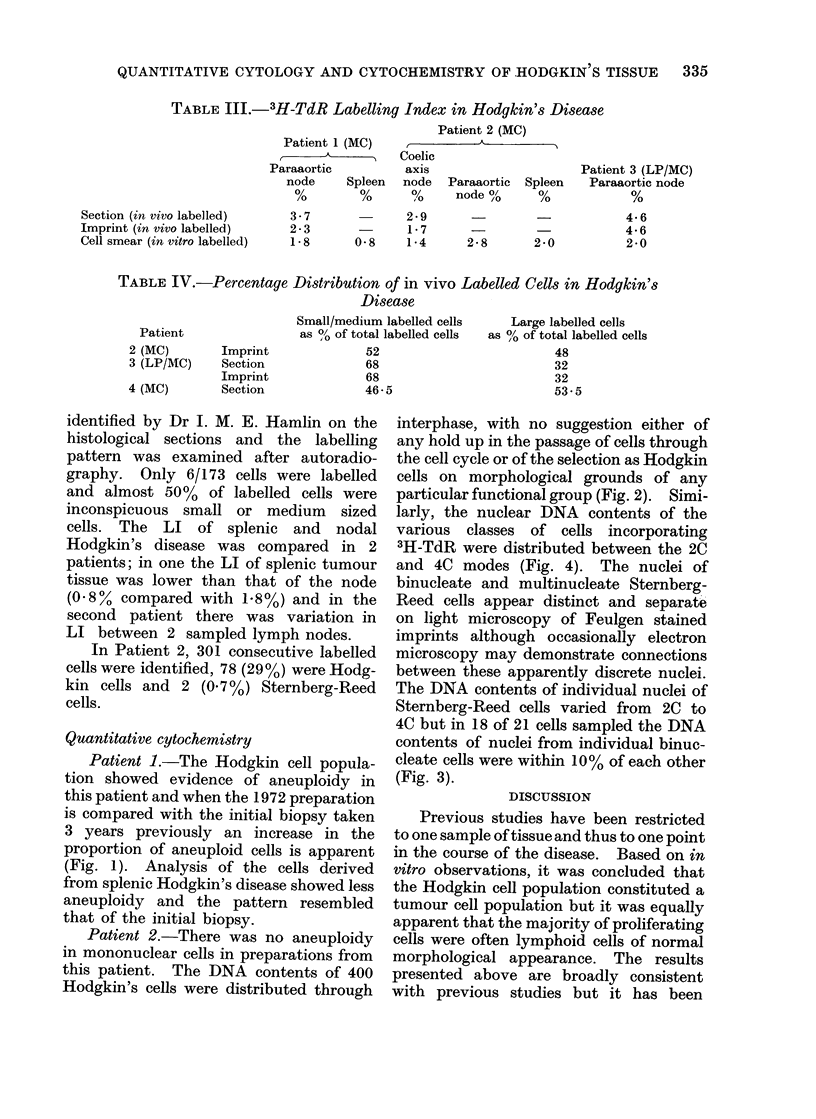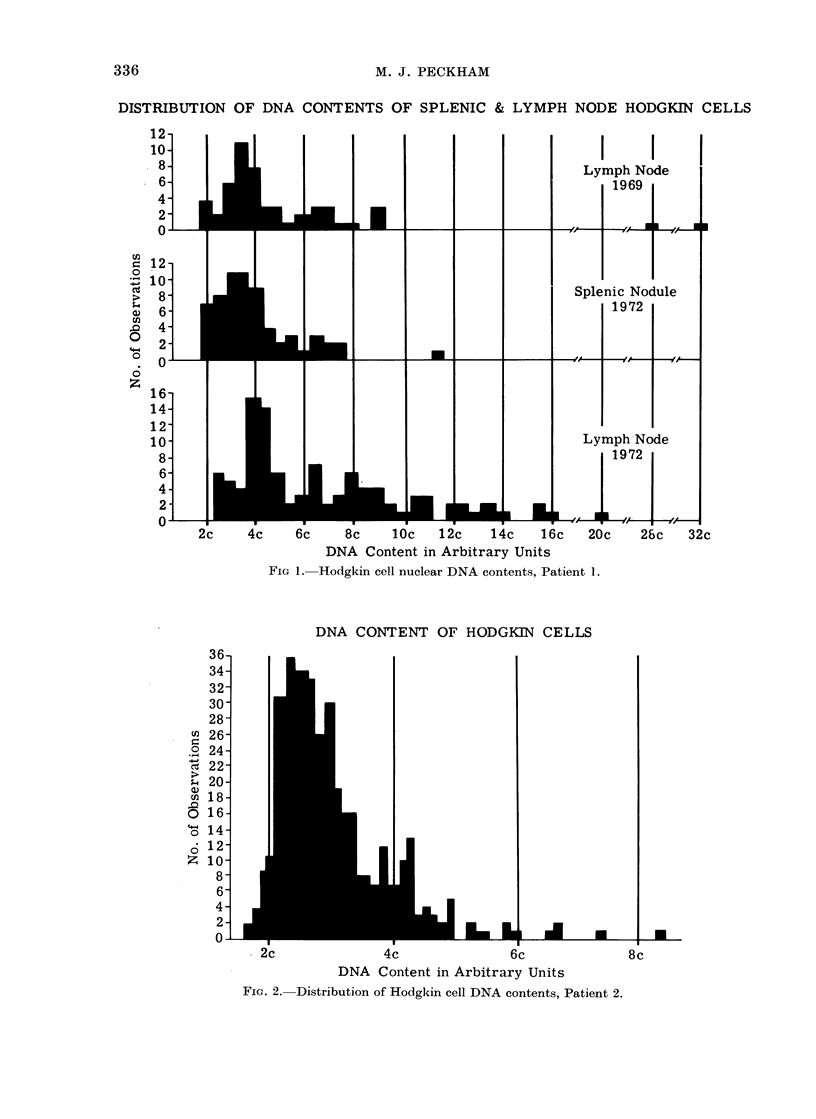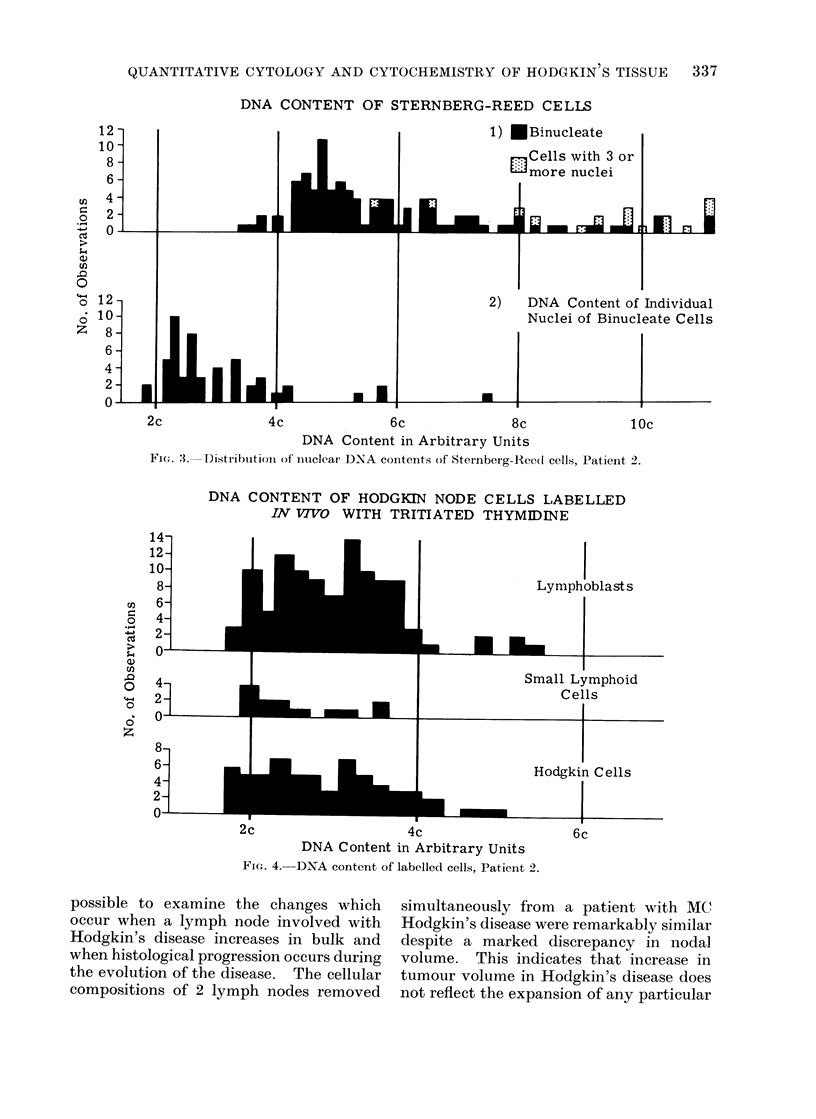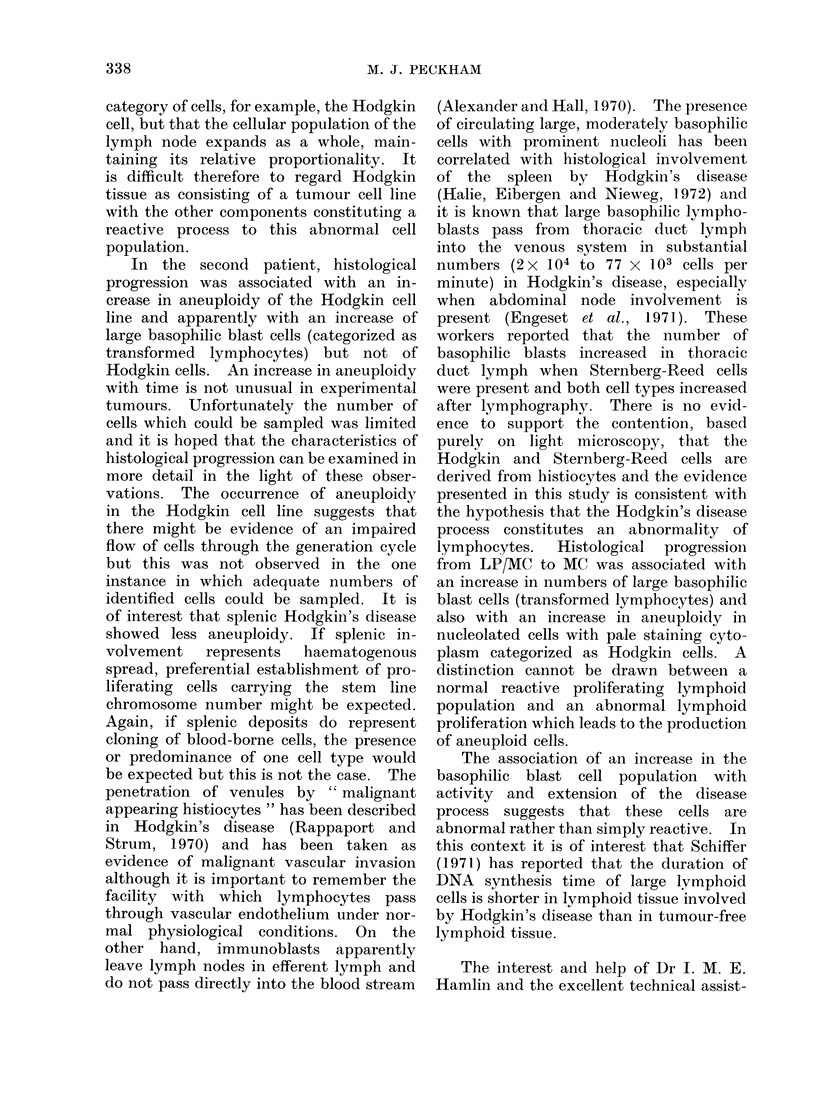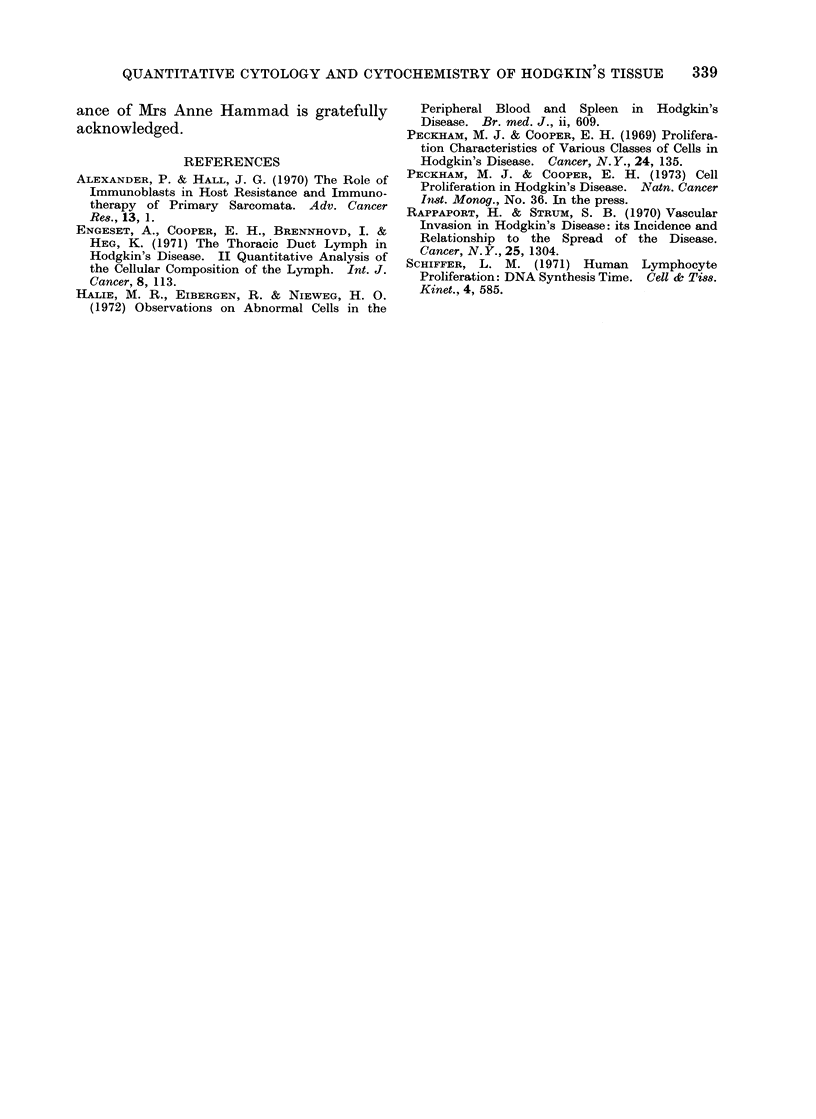# Quantitative Cytology and Cytochemistry of Hodgkin's Tissue Labelled in vivo with Tritiated Thymidine

**DOI:** 10.1038/bjc.1973.156

**Published:** 1973-10

**Authors:** M. J. Peckham

## Abstract

The cellular composition of Hodgkin's tissue from two patients has been examined. The labelling pattern with ^3^H-TdR has been studied on *in vivo* labelled imprints and histological sections. The relative proportions of the various classes of cells are maintained when Hodgkin's tissue increases in bulk. Histological progression from LP/MC to MC Hodgkin's disease was associated with an increase in aneuploidy of the Hodgkin cell line and an increase in the proportion of large basophilic blast cells. Splenic Hodgkin's disease in the same patient contained fewer aneuploid cells. The significance of these findings in terms of the histogenesis of Hodgkin and Sternberg-Reed cells is discussed.


Br. J. Cancer (1973) 28, 332

### QUANTITATIVE CYTOLOGY AND CYTOCHEMISTRY OF HODGKIN'S TISSUE LABELLED *IN VIVO* WITH TRITIATED THYMIDINE

M. J. PECKHAM

*From the Institute of Cancer Research, the Royal Marsden Hospital, London and Surrey*

Received 11 May 1973. Accepted 4 June 1973

**Summary.—The cellular composition of Hodgkin's tissue from two patients has been examined. The labelling pattern with $^3$H-TdR has been studied on *in vivo* labelled imprints and histological sections. The relative proportions of the various classes of cells are maintained when Hodgkin's tissue increases in bulk. Histological progression from LP/MC to MC Hodgkin's disease was associated with an increase in aneuploidy of the Hodgkin cell line and an increase in the proportion of large basophilic blast cells. Splenic Hodgkin's disease in the same patient contained fewer aneuploid cells. The significance of these findings in terms of the histogenesis of Hodgkin and Sternberg-Reed cells is discussed.**

A MORPHOLOGICAL distinction can readily be drawn between the tumour cell population and cells constituting the stroma in the majority of human tumours even when stromal elements are abundant. In Hodgkin's disease, however, there is a complex association of a variety of cell types, many of which appear normal morphologically, in contrast to the relative paucity in some cases of frankly atypical cells. Previous studies using a combined autoradiographic and cytochemical technique on cells derived from Hodgkin's lymph nodes labelled *in vitro* with tritiated thymidine ($^3$H-TdR) have shown that aneuploidy when present appeared to be confined to cells categorized as Hodgkin's cells (Peckham and Cooper, 1969). As many as 80% of the labelled cell population were small or medium sized cells apparently of the lymphoid series. A population of large, basophilic blast cells, often with a high labelling index, was identified and categorized on purely morphological grounds as transformed lymphocytes.

Detailed analysis of the cellular constituents of Hodgkin's tissue is limited by the infrequency of cells of the Sternberg-Reed, Hodgkin and transformed lymphocyte types in smears prepared from free cell suspensions. Imprints of Hodgkin's lymph nodes labelled *in vivo* by the direct injection of $^3$H-TdR allows a more satisfactory correlation of cell morphology, nuclear DNA content and uptake of isotopic tracer to be made (Peckham and Cooper, 1973). The opportunity to examine tissue taken from more than one site and the study of sequential biopsies, together with the application of *in vivo* labelling, allows the proportional distribution of the various categories of cells to be determined and related to both increase in bulk of the tumour mass and histological progression of the disease. In this study the relative proportions of the different morphological categories of cells have been estimated in lymph nodes and spleen removed from 2 patients. The cells have been examined as well as the labelling pattern on histological sections prepared from *in vivo* labelled tissue.

### MATERIAL AND METHODS

*Patient 1.*—This 49-year old male presented with Hodgkin's disease involving nasopharynx and cervical nodes in 1969. Histology was graded lymphocyte predominance/mixed cellularity (LP/MC). Lymphography was negative and he was clinically staged IIB. He remained well following

"mantle" irradiation until 1972 when a repeat lymphogram was positive. Laparotomy showed involvement of the spleen and liver as well as paraaortic lymph nodes. The material was graded mixed cellularity (MC). Cell suspensions from a cervical node (1969) were labelled *in vitro* with $^3$H-TdR. In 1972 a paraaortic node was labelled by direct injection and a splenic deposit by the *in vitro* technique.

*Patient 2.*—This 65-year-old male presented in 1971 with axillary and cervical involvement and was subjected to exploratory laparotomy and splenectomy. There was involvement of spleen, paraaortic and coeliac axis nodes and the material was graded MC. At laparotomy a large ($6 \times 3 \times 3$ cm) coeliac axis node was labelled by direct injection and a smaller paraaortic node ($2 \times 1 \times 1$ cm) as well as splenic deposit were labelled *in vitro*.

*Patients 3 and 4.*—In a third patient (MC) material labelled by direct injection of $^3$H-TdR was examined to compare the labelling index of the imprint with that of the section and to estimate the proportion of labelled small and medium lymphoid cells. An attempt was made in Patient 4 (LP/MC) to relate cell morphology identified by a histopathologist (Dr I. M. E. Hamlin) to labelling pattern on histological sections labelled *in vivo*. Only limited numbers of cells of diagnostic interest could be identified because of their low frequency.

*Labelling and preparation of autoradiographs.*—Techniques for *in vitro* labelling, the preparation of cell smears using a cytocentrifuge and subsequent autoradiography have been described previously (Peckham and Cooper, 1969). The details of *in vivo* labelling have also been described in another publication (Peckham and Cooper, 1973). In all cases photographic maps were made and cells identified after Giemsa staining. The cytological classification employed has been described in detail elsewhere (Peckham and Cooper, 1969, 1973). Briefly the following categories were recognized: small lymphocytes, plasma cells, eosinophils, polymorphs, histiocytes, small and medium labelled lymphoid cells, lymphoblasts (larger lymphoid cells with basophilic cytoplasm), transformed lymphocytes (large basophilic blasts, some with prominent nucleoli), Hodgkin cells (large cells with pale staining cytoplasm, lacy chromatin and large nucleoli) and Sternberg-Reed cells. Some cells were difficult to categorize as either Hodgkin cells or transformed lymphocytes and were termed intermediate cells.

*Labelling index.*—The overall labelling indices were obtained by counting a minimum of 2000 cells. Wherever possible the labelling index of individual cell types was obtained by counting 1000 cells, except in the case of low frequency cells such as Sternberg-Reed cells where a minimum of 200 cells was counted.

*Quantitative cytochemistry.*—Nuclear DNA contents were measured after Feulgen staining using a Deeley microdensitometer. Details are given elsewhere (Peckham and Cooper, 1969).

### RESULTS

### *Quantitative cytology*

*Patient 1.*—The cellular composition of the cervical node (LP/MC) was compared with that of the paraaortic node (MC) removed 3 years later. In the first preparation 36,000 cells and in the 1972 biopsy 100,000 cells were scanned and identified on photographic maps. Small lymphocytes accounted for 90% of the total cell population. Table I summarizes the findings. Table I(a) gives the total number of cells counted and Table I(b) the relative proportionality of lymphoblasts, transformed lymphocytes, Hodgkin cells, intermediate cells and Sternberg-Reed cells. In calculating the proportional distribution of cells, only cell types in which evidence of proliferation, as judged by the incorporation of $^3$H-TdR, have been included. Histocytes have been excluded since comparison of the numbers of esterase positive cells in cryostat sections with the numbers on imprints suggests that these do not separate readily from the fibroreticular framework of the node. There was an increase in the proportion of transformed lymphocytes and a reduction of lymphoblasts and Hodgkin cells in the 1972 specimen compared with the first node.

*Patient 2.*—Table II summarizes the results of the cellular composition of 2 nodes removed at laparotomy and differ-

TABLE I(a).—*Patient 1: Number of Cells Counted*

| Cell type | Cervical node (1969) LP/MC | Retroperitoneal node (1972) MC |
|---|---|---|
| Lymphoblast | 599 | 1256 |
| Plasma cell | 12 | 0 |
| Transformed lymphocyte | 22 | 1035 |
| Hodgkin cell | 77 | 160 |
| Intermediate cell | 5 | 71 |
| Sternberg-Reed cell | 5 | 1 |
| Histiocyte | 149 | 1578 |
| Mitotic figure | 2 | 3 |
| Polymorph | 487 | 464 |
| Total cells counted | 36000 | 100000 |

TABLE I(b).—*Patient 1: Percentage Distribution of Various Cell Types*

| Cell type | Node 1 (1969) LP/MC | Node 2 (1972) MC |
|---|---|---|
| Lymphoblast | 599 (84·6%) | 1256 (49·9%) |
| Transformed lymphocyte | 22 (3·1%) | 1035 (41%) |
| Hodgkin cell | 77 (18·9%) | 160 (6·3%) |
| Intermediate cell | 5 (0·7%) | 71 (2·8%) |
| Sternberg-Reed cell | 5 (0·7%) | 1 (0·04%) |
| Total | 708 (100%) | 2523 (100%) |

TABLE II(a).—*Patient 2: Number of Cells Counted*

| Cell type | Coeliac axis node (MC) (6 × 3 × 3 cm) | Paraaortic node (MC) (2 × 1 × 1 cm) |
|---|---|---|
| Lymphoblast | 579 | 727 |
| Plasma cell | 16 | 103 |
| Transformed lymphocyte | 176 | 79 |
| Hodgkin cell | 179 | 303 |
| Intermediate cell | 65 | 92 |
| Sternberg-Reed cell | 20 | 47 |
| Histiocyte | 197 | 291 |
| Mitotic figure | 4 | 28 |
| Polymorph | 17 | 126 |
| Total cells counted | 15000 | 15000 |

TABLE II(b).—*Patient 2: Percentage Distribution of Various Cell Types*

| Cell type | Coeliac axis node | Paraaortic node |
|---|---|---|
| Lymphoblast | 579 (56·8%) | 727 (58·7%) |
| Transformed lymphocyte | 176 (17·2%) | 79 (6·3%) |
| Hodgkin cell | 179 (17·5%) | 303 (24·5%) |
| Intermediate cell | 65 (6·4%) | 92 (7·8%) |
| Sternberg-Reed cell | 20 (1·9%) | 47 (3·9%) |
| Total | 1019 (100%) | 1248 (100%) |

ing in volume by a factor of about 20. The distribution of various cell types was similar in both nodes.

### $^3$*H-TdR labelling*

The overall labelling indices (LI) of the histological sections and imprints of the *in vivo* labelled material were compared with the *in vitro* LI (Table III).

The proportion of labelled cells tended to be lower than that of the sections in 2 patients but similar in the third patient. The histological sections and imprints of *in vivo* labelled material showed that a substantial proportion of labelled cells were small or medium sized lymphoid cells (46·5–68·0%) (Table IV).

In Patient 4, 173 large cells were

TABLE III.—$^3$*H-TdR Labelling Index in Hodgkin's Disease*

| | Patient 1 (MC) | | Patient 2 (MC) | | | Patient 3 (LP/MC) |
|---|---|---|---|---|---|---|
| | Paraaortic node % | Spleen % | Coelic axis node % | Paraaortic node % | Spleen % | Paraaortic node % |
| Section (*in vivo* labelled) | 3·7 | — | 2·9 | — | — | 4·6 |
| Imprint (*in vivo* labelled) | 2·3 | — | 1·7 | — | — | 4·6 |
| Cell smear (*in vitro* labelled) | 1·8 | 0·8 | 1·4 | 2·8 | 2·0 | 2·0 |

TABLE IV.—*Percentage Distribution of* in vivo *Labelled Cells in Hodgkin's Disease*

| Patient | | Small/medium labelled cells as % of total labelled cells | Large labelled cells as % of total labelled cells |
|---|---|---|---|
| 2 (MC) | Imprint | 52 | 48 |
| 3 (LP/MC) | Section | 68 | 32 |
| | Imprint | 68 | 32 |
| 4 (MC) | Section | 46·5 | 53·5 |

identified by Dr I. M. E. Hamlin on the histological sections and the labelling pattern was examined after autoradiography. Only 6/173 cells were labelled and almost 50% of labelled cells were inconspicuous small or medium sized cells. The LI of splenic and nodal Hodgkin's disease was compared in 2 patients; in one the LI of splenic tumour tissue was lower than that of the node (0·8% compared with 1·8%) and in the second patient there was variation in LI between 2 sampled lymph nodes.

In Patient 2, 301 consecutive labelled cells were identified, 78 (29%) were Hodgkin cells and 2 (0·7%) Sternberg-Reed cells.

*Quantitative cytochemistry*

*Patient 1.*—The Hodgkin cell population showed evidence of aneuploidy in this patient and when the 1972 preparation is compared with the initial biopsy taken 3 years previously an increase in the proportion of aneuploid cells is apparent (Fig. 1). Analysis of the cells derived from splenic Hodgkin's disease showed less aneuploidy and the pattern resembled that of the initial biopsy.

*Patient 2.*—There was no aneuploidy in mononuclear cells in preparations from this patient. The DNA contents of 400 Hodgkin's cells were distributed through interphase, with no suggestion either of any hold up in the passage of cells through the cell cycle or of the selection as Hodgkin cells on morphological grounds of any particular functional group (Fig. 2). Similarly, the nuclear DNA contents of the various classes of cells incorporating $^3$H-TdR were distributed between the 2C and 4C modes (Fig. 4). The nuclei of binucleate and multinucleate Sternberg-Reed cells appear distinct and separate on light microscopy of Feulgen stained imprints although occasionally electron microscopy may demonstrate connections between these apparently discrete nuclei. The DNA contents of individual nuclei of Sternberg-Reed cells varied from 2C to 4C but in 18 of 21 cells sampled the DNA contents of nuclei from individual binuccleate cells were within 10% of each other (Fig. 3).

### DISCUSSION

Previous studies have been restricted to one sample of tissue and thus to one point in the course of the disease. Based on *in vitro* observations, it was concluded that the Hodgkin cell population constituted a tumour cell population but it was equally apparent that the majority of proliferating cells were often lymphoid cells of normal morphological appearance. The results presented above are broadly consistent with previous studies but it has been

FIG 1.—Hodgkin cell nuclear DNA contents, Patient 1.

FIG. 2.—Distribution of Hodgkin cell DNA contents, Patient 2.

FIG. 3.—Distribution of nuclear DNA contents of Sternberg-Reed cells, Patient 2.

FIG. 4.—DNA content of labelled cells, Patient 2.

possible to examine the changes which occur when a lymph node involved with Hodgkin's disease increases in bulk and when histological progression occurs during the evolution of the disease. The cellular compositions of 2 lymph nodes removed simultaneously from a patient with MC Hodgkin's disease were remarkably similar despite a marked discrepancy in nodal volume. This indicates that increase in tumour volume in Hodgkin's disease does not reflect the expansion of any particular category of cells, for example, the Hodgkin cell, but that the cellular population of the lymph node expands as a whole, maintaining its relative proportionality. It is difficult therefore to regard Hodgkin tissue as consisting of a tumour cell line with the other components constituting a reactive process to this abnormal cell population.

In the second patient, histological progression was associated with an increase in aneuploidy of the Hodgkin cell line and apparently with an increase of large basophilic blast cells (categorized as transformed lymphocytes) but not of Hodgkin cells. An increase in aneuploidy with time is not unusual in experimental tumours. Unfortunately the number of cells which could be sampled was limited and it is hoped that the characteristics of histological progression can be examined in more detail in the light of these observations. The occurrence of aneuploidy in the Hodgkin cell line suggests that there might be evidence of an impaired flow of cells through the generation cycle but this was not observed in the one instance in which adequate numbers of identified cells could be sampled. It is of interest that splenic Hodgkin's disease showed less aneuploidy. If splenic involvement represents haematogenous spread, preferential establishment of proliferating cells carrying the stem line chromosome number might be expected. Again, if splenic deposits do represent cloning of blood-borne cells, the presence or predominance of one cell type would be expected but this is not the case. The penetration of venules by " malignant appearing histiocytes " has been described in Hodgkin's disease (Rappaport and Strum, 1970) and has been taken as evidence of malignant vascular invasion although it is important to remember the facility with which lymphocytes pass through vascular endothelium under normal physiological conditions. On the other hand, immunoblasts apparently leave lymph nodes in efferent lymph and do not pass directly into the blood stream (Alexander and Hall, 1970). The presence of circulating large, moderately basophilic cells with prominent nucleoli has been correlated with histological involvement of the spleen by Hodgkin's disease (Halie, Eibergen and Nieweg, 1972) and it is known that large basophilic lymphoblasts pass from thoracic duct lymph into the venous system in substantial numbers ($2\times 10^4$ to $77 \times 10^3$ cells per minute) in Hodgkin's disease, especially when abdominal node involvement is present (Engeset *et al.*, 1971). These workers reported that the number of basophilic blasts increased in thoracic duct lymph when Sternberg-Reed cells were present and both cell types increased after lymphography. There is no evidence to support the contention, based purely on light microscopy, that the Hodgkin and Sternberg-Reed cells are derived from histiocytes and the evidence presented in this study is consistent with the hypothesis that the Hodgkin's disease process constitutes an abnormality of lymphocytes. Histological progression from LP/MC to MC was associated with an increase in numbers of large basophilic blast cells (transformed lymphocytes) and also with an increase in aneuploidy in nucleolated cells with pale staining cytoplasm categorized as Hodgkin cells. A distinction cannot be drawn between a normal reactive proliferating lymphoid population and an abnormal lymphoid proliferation which leads to the production of aneuploid cells.

The association of an increase in the basophilic blast cell population with activity and extension of the disease process suggests that these cells are abnormal rather than simply reactive. In this context it is of interest that Schiffer (1971) has reported that the duration of DNA synthesis time of large lymphoid cells is shorter in lymphoid tissue involved by Hodgkin's disease than in tumour-free lymphoid tissue.

The interest and help of Dr I. M. E. Hamlin and the excellent technical assistance of Mrs Anne Hammad is gratefully acknowledged.

### REFERENCES

ALEXANDER, P. & HALL, J. G. (1970) The Role of Immunoblasts in Host Resistance and Immunotherapy of Primary Sarcomata. *Adv. Cancer Res.*, **13**, 1.

ENGESET, A., COOPER, E. H., BRENNHOVD, I. & HEG, K. (1971) The Thoracic Duct Lymph in Hodgkin's Disease. II Quantitative Analysis of the Cellular Composition of the Lymph. *Int. J. Cancer*, **8**, 113.

HALIE, M. R., EIBERGEN, R. & NIEWEG, H. O. (1972) Observations on Abnormal Cells in the Peripheral Blood and Spleen in Hodgkin's Disease. *Br. med. J.*, ii, 609.

PECKHAM, M. J. & COOPER, E. H. (1969) Proliferation Characteristics of Various Classes of Cells in Hodgkin's Disease. *Cancer, N.Y.*, **24**, 135.

PECKHAM, M. J. & COOPER, E. H. (1973) Cell Proliferation in Hodgkin's Disease. *Natn. Cancer Inst. Monog.*, No. 36. In the press.

RAPPAPORT, H. & STRUM, S. B. (1970) Vascular Invasion in Hodgkin's Disease: its Incidence and Relationship to the Spread of the Disease. *Cancer, N.Y.*, **25**, 1304.

SCHIFFER, L. M. (1971) Human Lymphocyte Proliferation: DNA Synthesis Time. *Cell & Tiss. Kinet.*, **4**, 585.